# Integrating temperature into the Arabidopsis circadian system

**DOI:** 10.1038/s44323-025-00055-z

**Published:** 2025-10-01

**Authors:** Chantal Sharples, Zoe Grace McFarlane, Maria Fernandes Pinheiro, Matthew Alan Jones

**Affiliations:** School of Molecular Biosciences, University of Glasgow, Glasgow, UK

**Keywords:** Circadian rhythms in plants, Plant sciences

## Abstract

This review discusses how temperature signals are integrated into the Arabidopsis circadian clock and proposes Temperature-Dependent Alternative Splicing (TDAS) of core clock genes as an additional mechanism to adapt the circadian system to temperature changes. We present examples of TDAS in a range of organisms, pointing towards a conserved mechanism that enables temperature adaptation.

## Temperature affects every biological process

Global warming is threatening ecosystems across the world and reshaping the balance of life. These impacts are far-reaching, ranging from declining marine biodiversity and rising animal extinction risks to reduced agricultural productivity^1,2^. Among the most vulnerable species are plants, whose sessile nature leaves them especially exposed to shifting climates. Increasing temperatures not only disrupt plant growth and development but also reduce exposure to low temperatures necessary for reproductive processes^3^. The impacts of climate change are evident at the molecular level, where temperature influences all aspects of protein biosynthesis. For example, temperature has been shown to cause differential protein expression in plants^4–6^ and to affect protein stability by disrupting protein folding and ligand binding^7,8^. These environmental changes make it imperative that we understand how plants perceive and respond to temperature, especially with regard to the circadian system that provides context to these environmental cues.

## Circadian systems are a ubiquitous concept found in a wide range of organisms

The Earth’s daily rotation has ensured that the majority of species possess an endogenous timekeeper which controls physical and behavioural changes in a repeating 24-h cycle^9,10^. This circadian system (or clock) is an intrinsic, molecular time keeping mechanism that coordinates cellular activity with daily environmental patterns, facilitating adaptation to changing environments^11^. Circadian rhythms were first observed in plants through leaf movements under constant light and were later linked to specific genes in *Drosophila melanogaster*, with their mammalian homologues revealing core clock components^12–14^. Although circadian systems differ between kingdoms and phyla, the genetic aspect of the circadian system has converged over evolutionary time to operate via interlinked Transcription-Translation Feedback Loops (TTFL) that drive rhythmic gene expression and circadian gating of biological events^12–15^. Metabolic circadian mechanisms have also been described; in the cyanobacterium *Synechococcus*, a post-translational circadian clock coordinates gene expression for photosynthesis during the day and nitrogen fixation at night via rhythmic phosphorylation of Kai proteins^16^. In eukaryotes, non-transcriptional oscillation in the form of circadian-driven redox cycles has been found in elements of the antioxidant system; these cycles are entrainable, offering an alternative or additional mechanism alongside the TTFL circadian system^17–19^.

The resetting of the circadian system to environmental cues ensures entrainment with dawn and dusk throughout the year while also allowing temperature compensation to increase the reliability of the biological timer as temperatures vary across daily and seasonal timescales. Reliable circadian timing enables emergent properties, including the external coincidence control of flowering and circadian gating, whereby molecular responses are modulated dependent upon time-of-day^9^. In this review, we assess how the Arabidopsis circadian system adapts to persistent temperature change and consider the consequences of global warming to plant circadian timing.

## The *Arabidopsis* circadian clock can be divided into three notional sub-groups

**Fig. 1:**
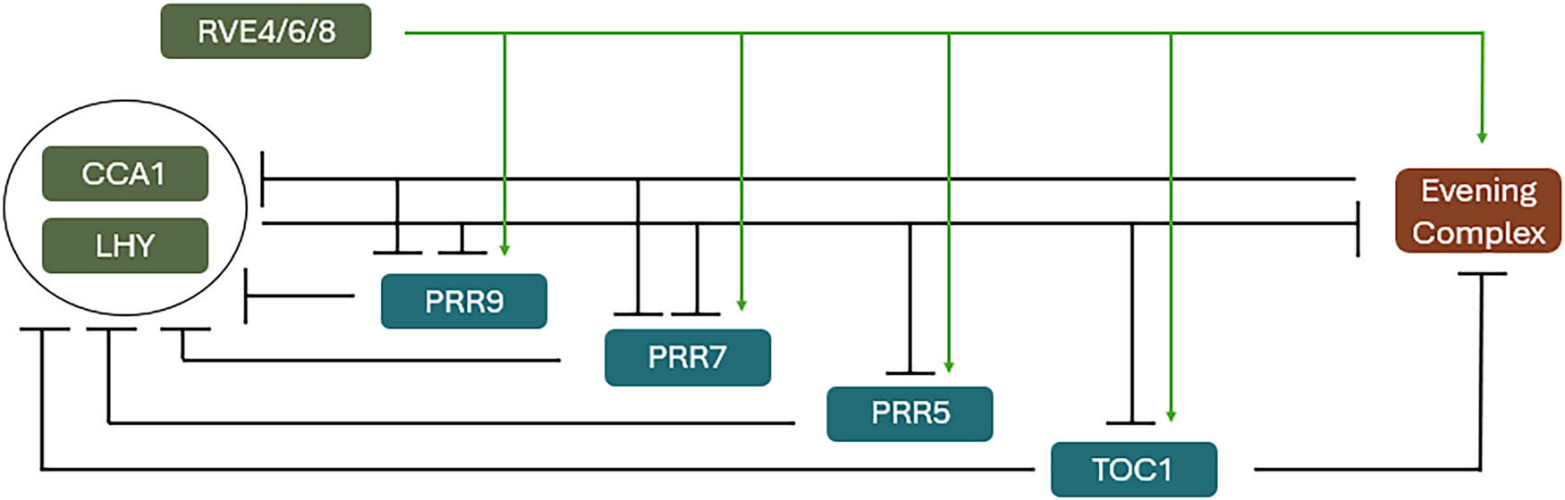
Outline of the Arabidopsis circadian TTFL. The plant circadian clock comprises at least three families of transcription factors that reciprocally regulate promoter activity, ultimately generating a biological timer with an approximate 24-h period. CIRCADIAN CLOCK ASSOCIATED1 (CCA1) acts with LATE ELONGATED HYPOCOTYL (LHY) to repress circadian gene expression immediately after dawn, whereas PSEUDO-RESPONSE REGULATORS (PRRs) repress circadian gene expression during the day. The Evening Complex, comprising LUX ARRHYTHMO, EARLY FLOWERING3 (ELF3), and ELF4 represses gene expression overnight to complete the sequential repression of circadian gene expression throughout the 24 h cycle. REVEILLE8 (RVE8) works as part of a complex to activate circadian gene expression in the morning.

Building upon the foundational observations of de Mairan, studies have described the botanic molecular oscillator to fit the classic TTFL model, exemplified in the model plant *Arabidopsis thaliana* (*Arabidopsis*, Fig. 1)^14,20–22^. Conceptually, the *Arabidopsis* circadian clock can be split into three temporally distinct groups: the Evening Complex (EC), the *PSEUDO-RESPONSE REGULATORS* (PRRs), and MYB-like transcription factors. The EC was the first identified circadian complex^23,24^: EARLY FLOWERING3 (ELF3) acts as a scaffold, interacting with ELF4 via its central domain and with LUX ARRHYTHMO (LUX) via its C-terminus^23–25^. ELF3 also binds phytochrome B (phyB), linking the circadian clock to light signalling^25^. Chromatin immunoprecipitation (ChIP) studies show that ELF3 and ELF4 bind more frequently to the promoters of genes like *PHYTOCHROME-INTERACTING FACTOR 4* (*PIF4*) and *PIF5* in the presence of LUX^23,26^. The interactions between ELF3, ELF4, and LUX are essential for the ECs' regulatory function^27^.

The MYB-like family in *Arabidopsis* includes core clock components such as *CIRCADIAN CLOCK ASSOCIATED 1* (CCA1) and *LATE ELONGATED HYPOCOTYL* (*LHY*), as well as the less-characterised *REVEILLE* (*RVE*) transcription factors *(RVE1-8)*, all sharing a conserved MYB-like DNA-binding motif (Fig. 1^28–31^). *CCA1* and *LHY* exhibit peak expression at dawn and ensure that the clock is synchronised with dawn^32^. Although disruption of *RVE1* function does not alter circadian timing, the other *RVE* genes have each been described to regulate timekeeping^31,33–36^. Like the EC, RVEs interact with various proteins to expand their molecular functions. For example, RVE8 associates with other RVEs (RVE4, 5, 6), TOLERANT TO CHILLING AND FREEZING1 (TCF1), a cold response regulator, and REGULATOR OF CHROMOSOME CONDENSATION1-LIKE (RCC1L)^37^. In the morning, RVE8 forms a complex with LIGHT NIGHT-INDUCIBLE AND CLOCK-REGULATED 1 (LNK1) and LNK2, transcriptional coactivators that influence gene expression (Fig. 1^34,37^). In the evening, COLD-REGULATED 27 (COR27) and COR28 join this complex to suppress RVE8 activity by regulating its abundance. Both CCA1 and RVE8 modulate *TOC1* expression via histone H3 modifications, with RVE8 promoting acetylation (associated with activation) and CCA1 inducing deacetylation (repression)^38,39^.

The PRR family is comprised of PRR5, PRR7, PRR9, and TIMING OF CAB EXPRESSION1 (TOC1), which function as a series of repressors expressed sequentially from mid-morning to dusk, following the peak of *CCA1* and *LHY* at dawn (Fig. 1^40–43^). They play a key role in negative feedback within the clock, as TOC1 indirectly promotes CCA1 by reducing its repressor, whereas CCA1 and LHY inhibit *TOC1* expression to form a feedback loop^44^. Like the other key clock families, PRRs form protein complexes to regulate gene expression. For instance, PRR5, PRR7, and PRR9 bind the promoters of *CCA1* and *LHY* via their C-terminal CONSTANTS, CONSTANS-LIKE, and TOC1 (CCT) domains, recruiting TOPLESS (TPL) proteins that help histone deacetylases repress *CCA1* and *TOC1* transcription^41^. This highlights the role of PRRs in maintaining clock stability and coordinating interactions between distinct components of the clock network.

## Temperature integration within the circadian system

Understanding how plants perceive temperature signals is critical for manipulating plant growth within our evolving environment. While a consensus exists regarding the broad aspects of light integration^45^, the identification of thermosensors is more complex as temperature changes generally influence all chemical reactions and many subcellular components. In alignment with photoreceptor definitions, we consider a biological thermosensor must directly translate the temperature stimulus into a biological signal via alteration of its own structure, activity, or interaction with other molecular components, triggering a downstream response^46^. In this context, ELF3, *PHYTOCHROME INTERACTION FACTOR 7* (*PIF7*) mRNA, and THERMO-WITH ABA-RESPONSE (TWA1), have been identified as thermosensors^47–49^. Additionally, photoreceptors, including phytochromes and phototropins have temperature-dependent activity that enables integration of light and temperature signals^50–52^.

**Fig. 2:**
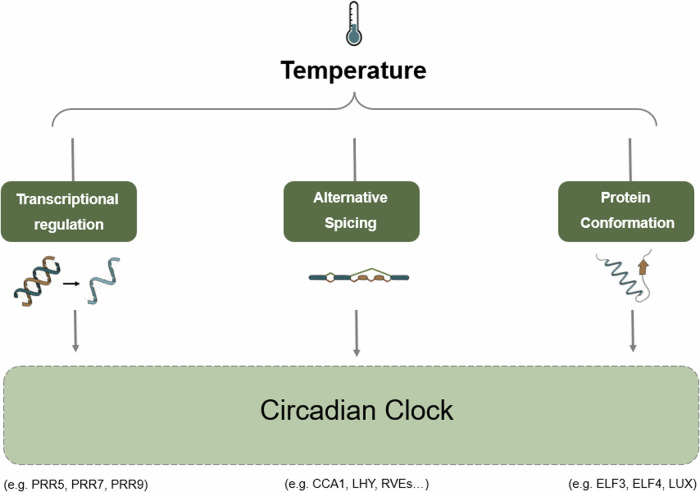
Temperature affects different aspects of the circadian system. The plant circadian clock comprises at least three families of transcription factors that reciprocally regulate promoter activity, ultimately generating a biological timer with an approximate 24-h period. Distinct mechanisms of temperature-modulated regulation have been characterised for certain genes within the circadian clock, although these are not mutually exclusive, and doubtless additional regulations will be elucidated over time.

Despite decades of research within circadian biology, the field continues to describe mechanisms by which light and temperature signals are integrated with the endogenous timing system (Fig. 2). Recent evidence highlights the role of the EC to integrate light and temperature signals. ELF3 undergoes conformational changes in response to temperature^47^. Increasing temperatures promote the accumulation of ELF3 into nuclear bodies via phase separation, preventing ELF3 from interacting with signalling partners^47^. The sequestration of ELF3 could enable integration of temperature information into the clock since the association of ELF3 with target gene promoters is temperature-dependent^53,54^. A temperature-dependent role for the EC is supported by the rescue of the *lux* arhythmic circadian phenotype at lower temperatures^55^. Alongside temperature signalling, phyB biological activity is also affected by temperature, with the half-life of the P_r_ form of phytochrome reduced as temperatures increase, allowing temperature to influence photoreceptor activity as part of the Evening Complex^25,50,52,56^.

Additional examples of the integration of light and temperature signalling arise from the regulation of PIF proteins. Phytochrome thermal reversion ensures PIF accumulation is temperature dependent^50,52,57^. PIF3 interacts with C-REPEAT BINDING FACTOR3 (CBF3) to stabilise phyB in response to cold temperatures, ultimately leading to increased freezing tolerance^58^. PIF4 and PIF5 have roles in heat stress responses, with PIF4 promoting growth and transition to flowering but decreasing immunity^59–61^. Circadian gating has also been linked to PIF4, suggesting that PIF4 thermosensitivity could contribute to the adaptation of the circadian system to temperature^62^. Furthermore, PIF7 translation is temperature sensitive, emphasising the pervasive effects of temperature upon biology^49,63^.

Beyond these proteins, numerous other temperature-dependent effects have been noted within the circadian system. Temperature-dependent phosphorylation of CCA1 by CASEIN KINASE2 alters circadian timing^64^, and temperature-dependent accumulation of circadian transcription factors and their interacting proteins has been reported. For example, RVE5 and RVE7 both accumulate at higher temperatures to alter gene expression^65,66^, while PRR7 and PRR9 contribute to temperature compensation^67^. Since TWA1 interacts with TOPLESS in a temperature-dependent manner, it will be interesting to determine whether TWA1 also contributes to temperature-dependent circadian gene expression via interactions with PRR proteins^41,48^. However, it remains unclear how temperature affects protein accumulation and activity in these cases.

## Alternative Splicing as a global thermometer

Alternative Splicing (AS), the process whereby a single gene generates multiple transcript isoforms through diverse combinations of exons and introns, contributes to proteomic diversity^68,69^. Isoforms exhibit diverse functionalities, with some contributing to distinct cellular processes while others may result in non-functional proteins due to the presence of premature termination codons, leading to RNA degradation via nonsense mediated decay (NMD)^68,69^. The presence of various isoforms alters the transcriptome and proteome, leading to a specific temperature-dependent response and enabling global changes in AS to provide thermosensitivity^6,70,71^. Temperature-dependent AS has been reported in commercially important crops such as tomato, rice, sugarcane, and barley, as well as *Arabidopsis*^71–77^. Indeed, this phenomenon is not restricted to plants but can also be found in yeast, fish, flies, and even mammals, pointing towards an evolutionary benefit of AS transmission of temperature signals^70,78–82^.

AS is facilitated by the spliceosome, a complex macromolecule composed of small nuclear ribonucleoproteins (snRNPs) designated U1-2, U4-6, and multiple ancillary proteins and RNA molecules^83^. Splicing factor recruitment by cis-regulatory sequences situated in exons and introns governs the selection of splice sites, leading to either activation or inhibition of splicing^83^. Since RNA structure is dependent on transcription rate, it appears plausible that interactions between spliceosome and ribosome activities enable temperature-dependent AS^49,84–87^. Alternatively, RNA riboswitches have been reported, with RNA structure changing in a temperature-dependent manner^87,88^. In either case, temperature-induced conformational changes within the spliceosome or RNA can modify the accessibility of splice sites, changing splicing outcomes^89^.

## Temperature-dependent AS of clock components

Several core components of the circadian clock, including *CCA1, LHY, PRR7, TOC1*, and *ELF3*, exhibit temperature-dependent AS, although the functional consequences of these alternative isoforms are mostly uncharacterised^71,73,90,91^. *CCA1* accumulates primarily in two isoforms: the full-length *CCA1α* and the truncated *CCA1β*, which are observed in comparable proportions at ambient temperature; however, a shift to *CCA1β* has been noted at elevated temperatures^92,93^. Whereas both isoforms compete for binding partners, only *CCA1α* encodes a DNA-binding protein, as it contains the MYB-like domain^94^. The presence of low temperatures affects AS of *CCA1*, promoting the accumulation of CCA1α^71,94^. Similarly, increases in canonical *LHY* transcript accumulation are observed at 12 °C compared to 20 °C, although this aligns with an increase in total *LHY* transcript levels. *LHY* transcript isoforms, including a 5′ UTR intronic retention, are elevated at 12 °C, although the biological function of this isoform compared to other isoforms remains to be determined^95^.

Temperature-dependent AS has not only been shown for *CCA1* and *LHY*, but also for *RVE2*. *RVE2* predominantly accumulates as an intron-retaining isoform at 20 °C, lacking translation of the functional MYB domains due to a premature stop codon^71^. Upon chilling, the canonical isoform becomes the most abundant. RVE2 has been demonstrated to influence the periodicity of the circadian clock and cold responses through the CBF signalling pathway^71^. Interestingly, RVE2 contributes to flowering time, although the temperature-dependent role has not been appreciated until recently^71,96^. It is possible that temperature-dependent AS of *RVE2* provides a signalling mechanism enabling ambient temperature to ‘re-program’ crucial developmental decisions.

## Future work prospects and conclusions

Recent discoveries are finally revealing specific mechanisms that define how plants respond to temperature and how these environmental cues are integrated into regulatory systems such as the circadian system. Temperature cues modulate the activity of circadian transcription factors to provide time-of-day context to environmental responses. However, the contribution of AS to circadian regulation has been limited by the lack of robust data detailing transcript isoform accumulation. Our review highlights the conserved mechanism of AS, which transmits temperature information and reprograms the transcriptome accordingly to the environmental changes, and theorises on the underlying causes that could promote AS in response to temperature. Enhanced comprehension of plant temperature signal perception will enable the manipulation of plant responses to the evolving environmental conditions driven by climate change.

## Data Availability

No datasets were generated or analysed during the current study.
